# Fetus in Fetu in the Scrotal Sac: Case Report and Literature Review

**DOI:** 10.1097/MD.0000000000001322

**Published:** 2015-08-14

**Authors:** Yi Ji, Bo Song, Siyuan Chen, Xiaoping Jiang, Gang Yang, Xia Gao, Bo Xiang

**Affiliations:** From the Division of Oncology (YJ, BS, XJ, GY, BX), Department of Pediatric Surgery; Pediatric Intensive Care Unit (SC); and Department of Pathology (XG), West China Hospital of Sichuan University, Chengdu, China.

## Abstract

Fetus in fetu (FIF) is a rare congenital anomaly. The most common site at which FIF occurs is the retroperitoneum. The mechanisms underlying the development of FIF have not been fully elucidated. The monozygotic twin theory postulates that FIF results from the unequal division of the totipotent cells of the blastocyst. However, the monozygotic twin theory does not explain all cases of FIF.

Herein, we describe the clinical characteristics of a 20-day-old infant with scrotal sac swelling. Ultrasonography and computed tomography revealed the presence of a mass consistent with a FIF rather than a teratoma. Surgical removal and a subsequent pathological evaluation demonstrated that the anencephalic fetus exhibited limb buds adjacent to a palpable vertebral column, supporting the diagnosis of FIF. The infant had an uneventful recovery and was discharged on the fifth postoperative day. In the present report, the pathogenesis, presentation, diagnosis, and management of FIF, as well as new concepts emerging in this area of research, are discussed.

Although the majority of cases of FIF may be diagnosed preoperatively, FIF should be distinguished from teratoma because the latter has substantial malignant potential. The recommended treatment for FIF is complete resection. To confirm the diagnosis of FIF, pathological examination, karyotyping, serologic marker assessment, and DNA restriction site mapping should be performed after removing the mass. Although FIF is thought to be a benign disorder, follow-up is necessary as a precaution against malignant recurrence, which has been described once.

## INTRODUCTION

Fetus in fetu (FIF) is a rare congenital anomaly, with an approximate incidence of 1 in 500,000 live births.^1^ Although FIF typically presents during infancy and early childhood, a small number of case reports of patients presenting with FIF during adulthood have been published. A limited number of cases of FIF have been detected prenatally as cystic intra-abdominal masses growing inside the fetus.^2^ The oldest patient with documented FIF was a 47-year-old man.^3^ To distinguish a FIF from a teratoma, Gunaydin et al^4^ described a FIF as a mass containing a vertebral axis with organs and limbs arranged around it. Interestingly, even on pathological examination, approximately 9% of FIF cases in the literature reviewed by Gonzalez-Crussi^5^ lacked a vertebral column. Gonzalez-Crussi^5^ subsequently stated that the diagnosis of FIF should be applied to any fetiform structure exhibiting significant organogenesis and the presence of an axial skeleton. The majority of FIFs occur in the retroperitoneal space. Scrotal FIF is extraordinarily rare. We describe a scrotal FIF preoperatively diagnosed and successfully operated.

## CASE REPORT

**FIGURE 1:**
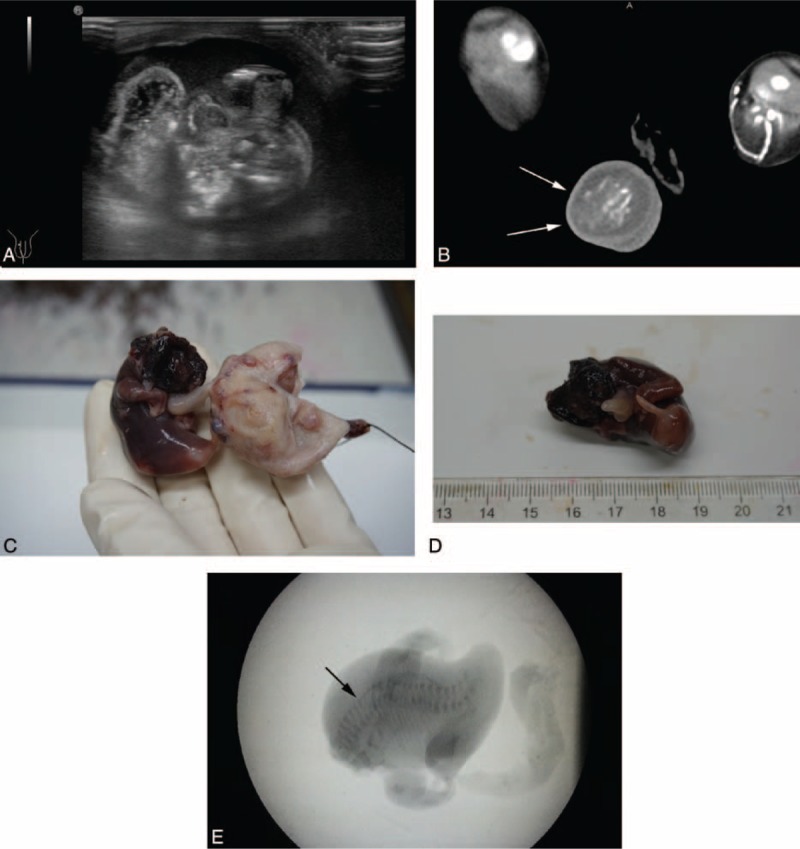
Scrotal FIF in a 20-day-old male neonate. (A) Ultrasound featuring a fetiform mass in the scrotal sac. (B) CT scan demonstrating a well-defined mass, measuring 4.5 × 4.0 cm, surrounded by a fluid-filled sac containing bony elements resembling the vertebral axis and the iliac bones (arrow head). (C and D) Upon incision, the mass exhibits a pinkish fetiform morphology, with tiny male external genitalia at one pole. (E) Plain radiograph of the FIF demonstrating vertebral organization (arrowhead). CT = computed tomography, FIF = fetus in fetu.

A 20-day-old infant was hospitalized for scrotal swelling following birth. The boy was born full term via a normal vaginal delivery, with a birth weight of 3250 g. There was no history of twinning or teratomas in either parents’ families. He had an enlarged scrotum, which contained a 4.5-cm firm mass. His complete blood count and kidney–liver function test results were within the respective reference ranges. A coagulation profile test showed the bleeding time, prothrombin time, activated partial thromboplastin time, fibrinogen level, and fibrin split products to be normal. Urinalysis values were normal. His β-human chorionic gonadotropin (β-HCG) and serum carcinoembryonic antigen (CEA) levels were also within the respective reference ranges. His serum α-fetoprotein (AFP) level was 1200 ng/mL. Ultrasonography of the scrotal sac demonstrated a well-defined fetiform mass measuring 3.5 × 2.0 cm, surrounded by a fluid-filled sac containing bony elements resembling a vertebral axis (Figure 1A). The left testis was normal in shape and size. The right testis, however, could not be found. A computed tomography (CT) scan revealed a well-encapsulated mass in which the fetal bone structures were visualized by ultrasonography (Figure 1 B). This finding was diagnostic of FIF; therefore, the boy was diagnosed with FIF.

**FIGURE 2:**
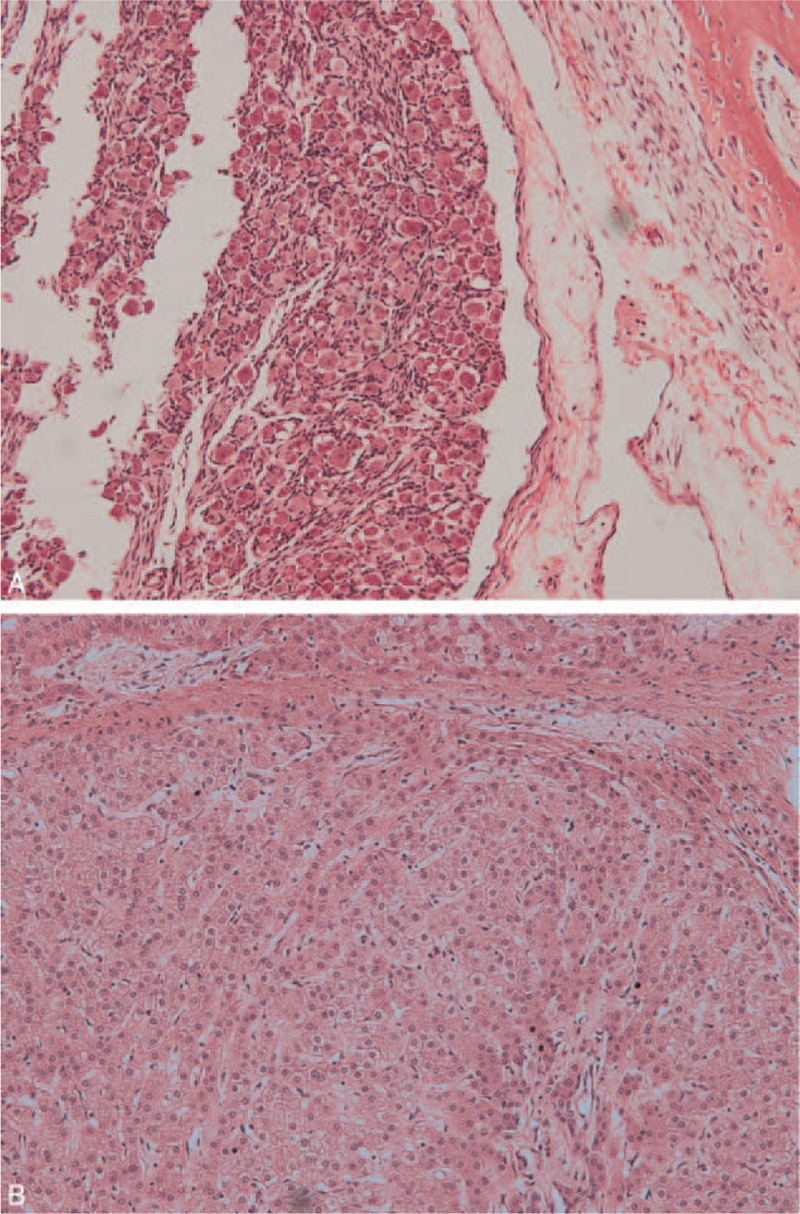
Histopathological examination revealed the presence of neural tissue, striated muscle tissue, and connective tissue (hematoxylin and eosin, ×200).

An incision in the right scrotal sac exposed a mass enveloped by a 4.5 × 4.0 × 3.0-cm capsule supplied by the right testicular artery. The capsule could be easily separated from the scrotal wall. The capsule was incised, and a skin-covered anencephalic FIF with a palpable vertebral column was subsequently noted. An umbilical cord-like structure was found to be contiguous with the capsule (Figure 1C). The FIF had 2 limbs that resembled hands with up to 7 digits. Tiny male external genitalia were clearly recognizable (Figure 1D). A specimen radiograph demonstrated a vertebral column (Figure 1E). Histopathologically, the FIF consisted of bone, cartilage, mucous glands, and neural and pancreatic tissue. A spinal cord with a central canal and adjacent vertebrae were found (Figure 2). The karyotype of the FIF was 46 XY. The patient's postoperative course was uneventful and he was discharged on the fifth postoperative day.

### Consent

Written informed consent regarding the publication of this case report and the accompanying images was provided by the patient's parents. Copies of the signed informed consent forms are available for review by the Series Editor of *Medicine*.

### Ethical Approval

This case report was approved by the Ethics Committee of the West China Hospital of Sichuan University, Chengdu, China. Written informed consent was obtained regarding the use of the images in accordance with the Declaration of Helsinki.

## DISCUSSION

FIF is an extremely rare condition, as <200 cases have been described in the literature worldwide. Since Hoeffel et al^6^ reviewed 87 cases of FIF in 2000, 89 additional cases have been published between 2000 and 2015. We have summarized the various sites at which FIF has been observed, as well as the details pertaining to the development of FIF outside of the abdomen (Table 1). The most common site of FIF is the retroperitoneum (75.6%); atypical locations include the skull (9%), mediastinum, thorax, sacrum, scrotum, mouth, neck, pelvis, and liver. The sex ratio (male to female) of FIF is approximately 1.5:1. Even under pathological examination, only 84.3% of cases of FIF have been confirmed to have a vertebral column.

**TABLE 1:**
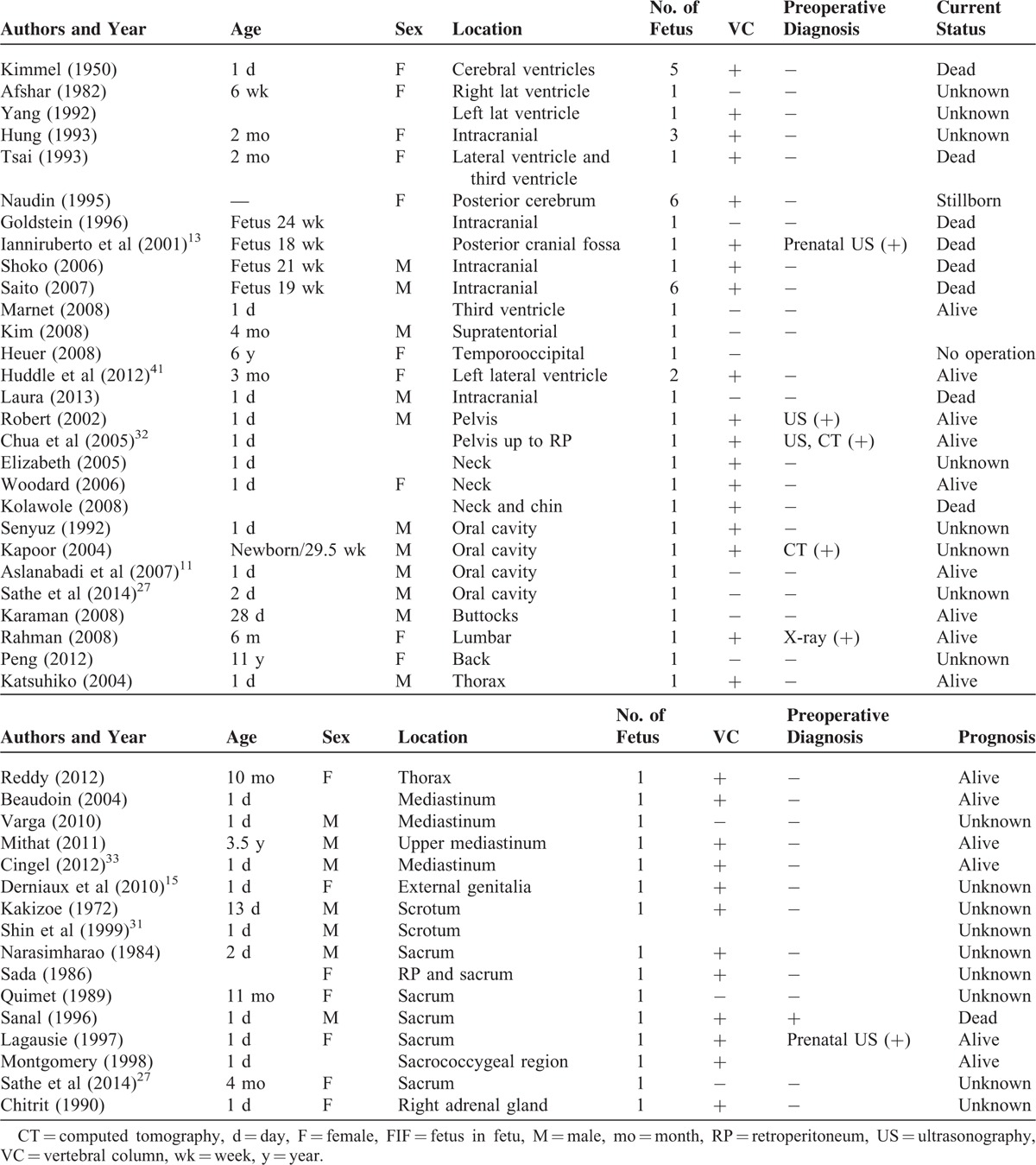
Published Cases of FIF With the Exception of Those in the Abdomen and Retroperitoneum

### Pathogenesis

A FIF is a malformed fetiform mass located within a host, defined as an encapsulated, pedunculated, vertebrate, fetiform mass.^7^ The definition devised by Gunaydin et al^4^ requires the presence of an axial skeleton and appears to be too strict. The presence of vertebral bodies indicates that the FIF progressed through the primary stage of gastrulation. The neural tube develops before the skeleton, including the spinal column, and is important in the development of other structures; therefore, the absence of a spinal column at term may not preclude the possibility that one existed at an earlier stage.^8^ The cases of FIF characterized by the absence of a vertebral column are also often characterized by identifiable limbs and well-formed long bones, which must initially have been shaped via the influence of innervated muscles.^9^

A FIF is typically enclosed within a distinct sac containing either fluid or sebaceous material. An umbilical cord-like structure serves as the connection between the fetus and the host. FIFs are usually both cardiac and anencephalic. In Spencer's opinion, the absence of the heart is not conducive to growth or the sustenance of life, which may explain the growth retardation and arrest of organ differentiation observed in the setting of FIF.^7^ However, there have been cases of FIF in which a heart was present,^10–13^ including one in which a heart rate of 108 beats/min was recorded.^12^

The pathogenesis of FIF remains controversial. Most investigators agree with the monozygotic twin theory, which postulates that FIF results from the unequal division of the totipotent cells of a blastocyst. According to the monozygotic theory, a FIF is a diamniotic, monochorionic, monozygotic twin that becomes incorporated into the body of a host twin following the anastomosis of the vitelline circulation.^14^ However, the monozygotic, monochorionic, diamniotic twin theory may not explain all cases of FIF, including cases characterized by the presence of multiple fetuses associated with a single host or a FIF associated with a teratoma.^15–17^ The monozygotic, dichorionic, diamniotic twin theory may supply the answers regarding these phenomena. At 30 hours following fertilization, the zygote consists of 2 cells. Spontaneous division of the zygote into 2 embryos is not considered a hereditary trait but rather a spontaneous event. If the zygote splits early (within the first 2 days following fertilization), the embryos may develop separate placentas (chorion) and separate sacs (amnion). These embryos are dichorionic, diamniotic twins. On approximately the fifth day of the development, the blastocysts implant into the uterine endometrium; however, if one of the blastocysts derived from the conceptus implants into the other blastocyst instead of the endometrium, or if one blastocystis enclosed by the other implant close together, the subsequent development of such an inclusion body may result in FIF. From an embryological point of view, the existence of avertebral column in a fetiform mass may reflect its derivation from the primitive streak. The formation of the primitive streak normally begins during the third week of development in conjunction with gastrulation, which results in notochord formation and the subsequent development of the vertebral column and segmental axis. Therefore, a FIF may initially develop in a manner similar to that of normal fetal development. Interestingly, studies of genetic markers and genotyping suggest that host infants and their fetiform masses are genetically identical, which supports the monozygotic twin theory.^18–21^

As mentioned previously, the monozygotic twin theory does not explain all cases of FIF, and the theory surrounding a possible relationship between FIF and a highly differentiated teratoma remains controversial. Some investigators have hypothesized that a FIF represents a well-differentiated and highly organized teratoma.^22^ In other words, FIFs and teratomas may share an underlying pathogenetic mechanism. Proponents of the teratoma theory argue that FIFs may lack a spinal column and that teratomas may be highly differentiated and organogenic. Highly differentiated teratomas may contain a variety of organs, such as well-formed limbs, teeth, intestinal loops, a spinal cord, and brain-like tissue.^8^ FIFs may be found at many of the sites at which teratomas are located, including the retroperitoneum and ovaries. FIFs may be associated with teratomas, and retroperitoneal teratoma formation has been described following FIF removal.^12,23^ As there are many similarities between the 2 entities at the histological level, attaining a full understanding of the true nature of a FIF is an essential step toward improving treatment efficiency in the setting of this rare disease.

### Presentation

Most cases of FIF involve a single parasitic twin. However, >1 parasitic twin may be observed in the host body, although such occurrences are rare. To the best of our knowledge, the maximum number of FIFs documented previously is 11.^24^ Eighty-nine percent of FIF lesions have been noted before 18 months of age.^6^ Most FIFs are located within the retroperitoneal space^25^; additional sites include the cerebral ventricles,^26^ mouth,^11,27^ neck,^28^ adrenal gland,^29^ liver,^30^ scrotum,^31^ pelvis,^32^ and mediastinum.^33^ As FIF is a benign disorder and has been described at various sites ranging from the cranial cavity to the scrotal sac, FIF lacks specific symptoms. Most FIFs present as asymptomatic, slow-growing abdominal masses. The symptoms of FIF are usually related to mass effect and include abdominal distension, feeding difficulties, emesis, jaundice, and dyspnea, which are caused by the compression of adjacent organs and tissues. If an FIF develops in the cranial cavity, neurologic symptoms may be observed.

### Diagnosis

To diagnose FIF, one of the following characteristics must be present: a mass enclosed within a distinct sac, either partially or completed covered by skin, with grossly recognizable anatomic features attached to the host via a pedicle containing a small number of relatively large blood vessels.^34^ Both ultrasonography and plain radiography may be useful in identifying these features. Different organs may be observed within a FIF, including limbs (86.0%) and a vertebral column (84.3%). Other organ systems include the central nervous system, gastrointestinal tract, blood vessels, and genitourinary tract. A FIF may be differentiated from a teratoma by the presence of vertebral organization with the limb buds and the other organ systems.

Ultrasonography may be helpful in the diagnosis of FIF as early as 21 weeks of gestation. It was first used by Nicolini et al^35^ to make a prenatal diagnosis of FIF. Ultrasonography may reveal the presence of calcific structures, but it may also result in misdiagnosis. Calcifications within the fetal abdomen may be caused by a teratoma, meconium peritonitis, a neuroblastoma, viral infections, and adrenal hemorrhage.^36^ Careful scrutiny of the calcifications, as well as magnetic resonance imaging (MRI), may enable clinicians to make a correct diagnosis. When fetal ultrasonography cannot distinguish between a cyst and a solid mass, fetal MRI may be useful.

Ultrasonography and x-ray imaging may also aid in diagnosing FIF, as both modalities may help clinicians to visualize various aspects of the fetal skeleton, including the vertebral column. CT and MRI may allow for a more accurate diagnosis and may help define the relationship between the FIF and any adjacent intra-abdominal structures (Figure 1). In patients with a FIF, CT and MRI may reveal the presence of a distinct gestational sac containing diverse fetal structures (Figure 2). Distinct bony structures, such as the spine and the bones of the extremities, may be visualized using CT and MRI, both of which have enhanced the accuracy of preoperative diagnoses.^6,37^

Complete blood counts and kidney–liver function tests are frequently performed, and the results are usually within reported reference ranges. To distinguish a FIF from a malignant teratoma, both serum β-HCG and serum AFP levels should be tested. Serum AFP is an important indicator of the level of the malignancy of the teratoma, and elevated serum β-HCG levels may reflect either the presence of chorionic tissues within the mass or teratoma recurrence. Testing serum CEA levels may help clinicians and researchers to exclude specific types of abdominal tumors. The results of the serological examinations mentioned above may be helpful in making a preoperative diagnosis of FIF.

Despite the above-mentioned tests, making a definitive diagnosis preoperatively is often difficult. Postoperative pathological examinations, genetic marker assessment, and genotyping should be undertaken to confirm the diagnosis. Under gross pathological examination, various well-differentiated organs may be observed, including the vertebral column, limbs with digits, and other organs. Approximately 15.7% of FIF cases lack a vertebral column; however, such cases must have the aforementioned characteristics to be consistent with a diagnosis of FIF. Even if one mass with a well-differentiated axial skeleton is confirmed, the diagnosis of FIF may not be 100% certain. Kuno et al^38^ described a fetiform teratoma with a highly developed axial skeleton that was distinguished from a FIF on the basis of zygosity.

### Differential Diagnosis

#### Teratoma

Teratomas are solid tumors that are common in infancy and are composed of various tissues foreign to the sites at which they arise.^7^ They are embryonal tumors that develop abnormally from the 3 primitive blastoderm cell layers. They may contain parts of or each of the 3 germ cell layers, with different degrees of maturity. The tissue types most often observed in highly differentiated teratomas are epithelium, hair, cerebral tissue, neurocytes, cartilage, bone, and teeth; however, teratomas lack an axial skeleton. However, because some FIFs lack a spinal column, some investigators have suggested that FIFs area type of highly differentiated teratoma.^22^ Making the diagnosis may be difficult in these cases because of the similarities in the entities’ clinical and radiological features, as well as in their appearances on histological examination. The most common sites are the sacrococcyx, anterior mediastinum, testicles, ovaries, and retroperitoneum, which are also sites at which a FIF may be observed. The incidence of teratomas is approximately 1/4000 live births,^39^ which is much higher than that of FIF. Approximately 7% of teratomas are malignant, whereas FIF is a benign disease. Although the pathology results may be similar between FIF and teratomas,^15^ karyotyping, serologic marker assessment, and DNA restriction site mapping may confirm that the mechanism underlying the development of FIF is different than that underlying the development of a teratoma. Regarding fetiform teratomas, which may have a spinal column, the most common location is the ovaries. This entity may be differentiated from a FIF via zygosity, as most fetiform teratomas are homozygous, and FIFs are heterozygous. Additionally, fetiform teratomas are most commonly found in women of reproductive age, whereas FIF is most often observed during infancy.^6,40^

#### Meconium peritonitis

Meconium peritonitis is the result of sterile chemical peritonitis, which results from an intrauterine bowel perforation and the subsequent exudation of meconium into the peritoneal cavity. The most common radiographic finding in newborns with meconium peritonitis is calcification, which is visualized as a classic eggshell calcification. Other x-ray imaging findings include ascites and pneumoperitoneum, which may also be observed in FIF. The calcifications associated with FIF, which most often resemble bony structures, are often used to distinguish a FIF from meconium peritonitis.^36^ The differential appearances of these diseases on imaging studies are presented in Table 2.

**TABLE 2:**
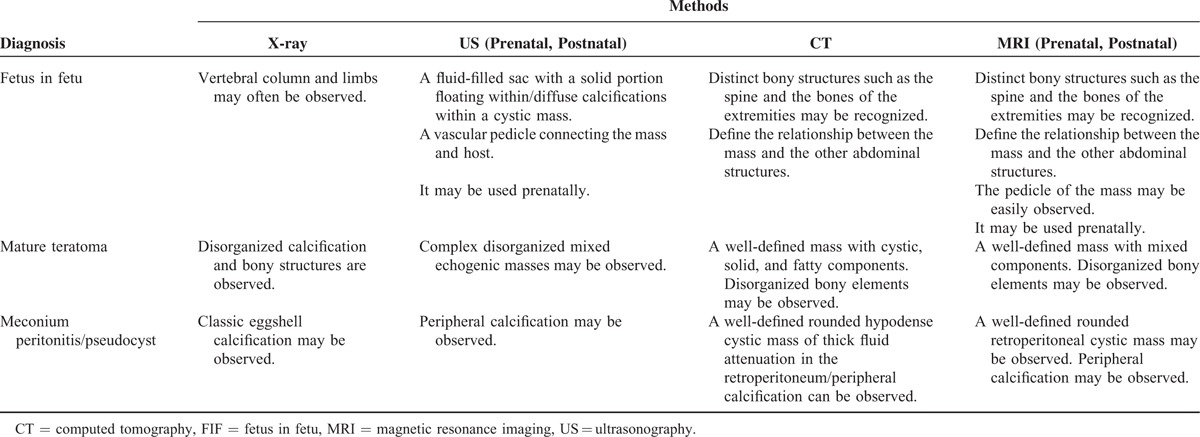
Imaging Findings Pertaining to the Differential Diagnosis of FIF

### Management

Although FIF is thought to be a benign disorder, the recommended treatment for FIF is surgical excision to relieve obstruction, prevent further compression, and minimize the risk of hemorrhage. Significant blood loss during surgery is common; therefore, packing the abdomen may be helpful in achieving hemostasis for cases involving either the abdomen or the retroperitoneum. The resection of all parts of the mass is necessary because the diagnosis of FIF cannot be confirmed until a pathological analysis has been performed. Additionally, removing the entire mass may also decrease the risk of local malignant recurrence.

The prognosis of most cases of FIF is good, although the prognosis of intracranial FIF is poor. Only 2 of the 18 patients with an intracranial FIF have survived, 1 of whom has only minimal head control and suffers from spasticity in the lower extremities.^41^ The indicators for postoperative follow-up include serum levels of β-HCG and AFP, which are suggestive of the development of malignant teratoma. Although some cases of FIF are associated with high CEA levels,^14^ there is no evidence of a correlation between FIF and CEA. Either CT or MRI should also be performed at 3, 6, 12, and 24 months following therapy.

## CONCLUSIONS

FIF is a rare entity that typically presents during infancy and early childhood. Current imaging modalities enable more accurate diagnosis and define the relation of the FIF with the other structures before surgery. FIF should be differentiated from a teratoma because of the malignant potential of the latter. Although the prognosis of FIF is generally good, cases of malignant degeneration have been described. Clinical and iconographic follow-up are therefore encouraged.
